# Digital Pills with Ingestible Sensors: Patent Landscape Analysis

**DOI:** 10.3390/ph15081025

**Published:** 2022-08-19

**Authors:** Olena Litvinova, Elisabeth Klager, Nikolay T. Tzvetkov, Oliver Kimberger, Maria Kletecka-Pulker, Harald Willschke, Atanas G. Atanasov

**Affiliations:** 1 Department of Management, Economy and Quality Assurance in Pharmacy, National University of Pharmacy, The Ministry of Health of Ukraine, 61002 Kharkiv, Ukraine; 2 Ludwig Boltzmann Institute Digital Health and Patient Safety, Medical University of Vienna, 1180 Vienna, Austria; 3 Institute of Molecular Biology “Roumen Tsanev”, Department of Biochemical Pharmacology and Drug Design, Bulgarian Academy of Sciences, 1113 Sofia, Bulgaria; 4 Department of Anaesthesia, Intensive Care Medicine and Pain Medicine, Medical University of Vienna, 1090 Vienna, Austria; 5 Institute for Ethics and Law in Medicine, University of Vienna, 1090 Vienna, Austria; 6 Institute of Genetics and Animal Biotechnology of the Polish Academy of Sciences, 05-552 Jastrzebiec, Poland

**Keywords:** digital pill, ingestible sensor, patent, clinical monitoring, medication adherence

## Abstract

The modern healthcare system is directly related to the development of digital health tools and solutions. Pills with digital sensors represent a highly innovative class of new pharmaceuticals. The aim of this work was to analyze the patent landscape and to systematize the main trends in patent protection of digital pills with ingestible sensors worldwide; accordingly, to identify the patenting leaders as well as the main prevailing areas of therapy for patent protection, and the future perspectives in the field. In July 2022, a search was conducted using Internet databases, such as the EPO, USPTO, FDA and the Lens database. The patent landscape analysis shows an increase in the number of patents related to digital pills with ingestible sensors for mobile clinical monitoring, smart drug delivery, and endoscopy diagnostics. The leaders in the number of patents issued are the United States, the European Patent Office, Canada, Australia, and China. The following main areas of patenting digital pills with ingestible sensors were identified: treatment in the field of mental health; HIV/AIDS; pain control; cardiovascular diseases; diabetes; gastroenterology (including hepatitis C); oncology; tuberculosis; and transplantology. The development of scientific and practical approaches towards the implementation of effective and safe digital pills will improve treatment outcomes, increase compliance, reduce hospital stays, provide mobile clinical monitoring, have a positive impact on treatment costs and will contribute to increased patient safety.

## 1. Introduction

Nowadays, in the modern world the progress of the healthcare system is directly related to the development of digital health tools.

According to the WHO global strategy, digital technologies are connected to the future of world health. Digitalization has the potential to benefit health promotion, maintain global security, and provide services to the most vulnerable groups of the population [1].

Digital pills occupy an important place among the digital health solutions. Digital pills contain integrated sensors that allow monitoring of the course of pharmacotherapy through an interaction with the software of, e.g., tablets and smartphones. Such monitoring is of great importance, as low patient compliance (medication opt-out) is a major challenge for all areas of medicine.

Digital pills improve treatment adherence and efficiency in the field of mental health and behavioral modifications, such as schizophrenia, bipolar I disorder, attention deficit and hyperactivity disorder, drug abuse, smoking, pain, insomnia, and many others. The developers of the digital pills also focus on the treatment of cardiac disorders, diabetes, hepatitis C, AIDS, cancer, tuberculosis, and the monitoring of patients’ use of opioid drugs after surgery, and other conditions when admission may be impaired due to the characteristics of the patient’s behavior (geriatrics, neurodegenerative diseases, etc.) [2,3,4].

Digital pills have a significant potential for savings in healthcare costs by reducing the need for emergency medical care and the hospitalization of patients. The annual costs of non-compliance range from USD100 billion up to USD290 billion in the US, EUR1.25 billion in Europe, and approximately USD7 billion in Australia. In addition, 10% of the hospitalizations among the elderly are due to treatment noncompliance, with a typical noncompliant patient requiring three additional doctor visits per year, resulting in an annual increase of USD2000 in treatment costs. In diabetes, the estimated cost savings associated with improving noncompliance ranges from USD661 million to USD1.16 billion. Non-adherence is thus a critical clinical and economic problem [5]. 

Despite the progress made in this area to date, there are still a number of barriers to the widespread implementation of digital pills into medical practice. They include issues of clinical efficacy, safety, treatment costs, and confidentiality, among others. In addition, the patent landscape for the digital pill with ingestible sensors is not yet well-established. This indicates the need for further research in this area [6,7].

The development of digital pills is executed by high-tech industries that are evolving rapidly and require innovation from manufacturers. One of the sources of information reflecting the innovation process is the patent documentation.

The value of information, which is formed as a result of the work of patent offices in different countries around the world, is its universality in determining the main technological trends and building trends in market processes, and in analyzing the behavior of specific market participants, their resources, and growth prospects. The universality of patent data is ensured by the unification of standards for the presentation of data on intellectual property objects. The reliability of patent information is ensured by the procedure of the state registration of intellectual property rights. The scope of their legal protection depends on the completeness of the disclosure of information about the objects, as well as on the concretization of the features that constitute the novelty of the results of intellectual property. Therefore, in order to ensure a comprehensive protection of their own exclusive rights, the applicant is forced to detail the important technological aspects of patented development as much as possible. The examination of the patent landscape enables researchers to quantify the intellectual property characteristics.

The aim of this work was to analyze the patent landscape and systematize the main trends in the patent protection of digital pills with ingestible sensors worldwide, as well as to identify the patenting leaders, the main prevailing areas of therapy for patent protection, and future perspectives in the field.

## 2. Results and Discussion

By the end of June 2021, there were 137 digital therapeutic products and 122 digital care products at different stages of development, according to the IQVIA Digital Solutions database, which systematizes the different types of digital healthcare solutions [8]. Among the so-called Digital Therapeutics, 25 had secured market authorization and became available for marketing through regulatory processes. Among these 25 Digital Therapeutics with market authorization from at least one country, 9 were in the US, 19 were in Europe, and 1 was in Japan, with some overlap [8].

**Figure 1:**
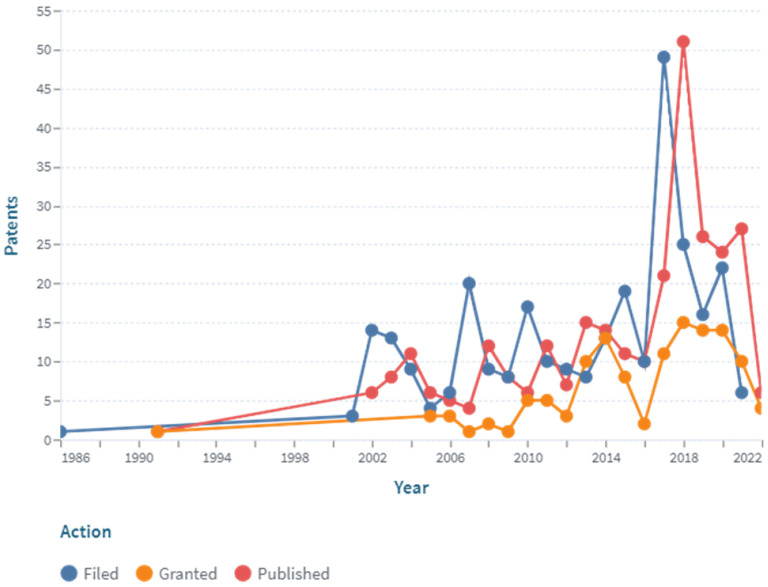
Dynamics of patent activity by applicants in the field of digital pills with ingestible sensor (https://www.lens.org, accessed on 30 July 2022).

An analysis and systematization of the global patent protection of digital pills with ingestible sensors was completed, to identify the key developments in the sector (Figure 1).

A stable increase in the dynamics of applications and granted patents during the period of time between 2010 and 2018 was revealed. Most of the applications (49) were filed in 2017, and most of the patents (15) were granted in 2018. The lower patent activity in the years 2019–2022 (the first half of the year) may be associated with a focus on commercialization and the entry into the market of newly created inventions instead of the filing of new patents.

**Figure 2:**
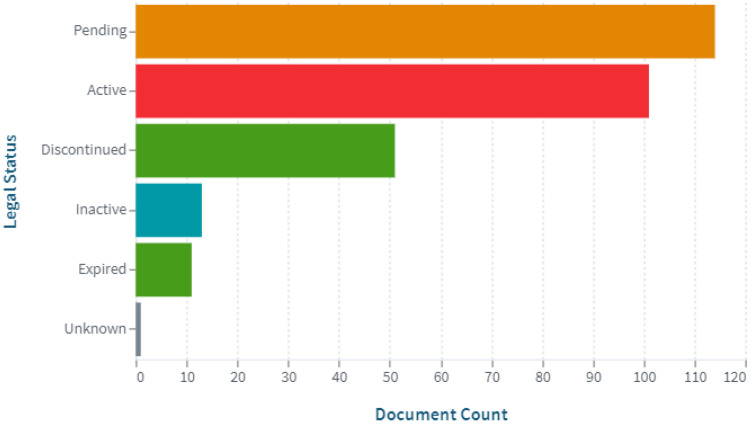
Patent documents by legal status (https://www.lens.org, accessed on 30 July 2022).

The patenting landscape presents a strong indication of the continuous efforts of scientists in the development of digital pill forms. It should be noted that some of the patents are no longer valid (Figure 2). Given the active development and replacement of technologies with newer, more modern ones, the patents are rarely kept in force for the maximum of 20 years (25 years for medicines). Most of the analyzed patents were filed during the period between 2000 and 2018. 

The leaders in the number of patents issued are the United States, the European Patent Office, Canada, Australia, and China, which together account for 72% of the total number of patents around the world.

**Figure 3:**
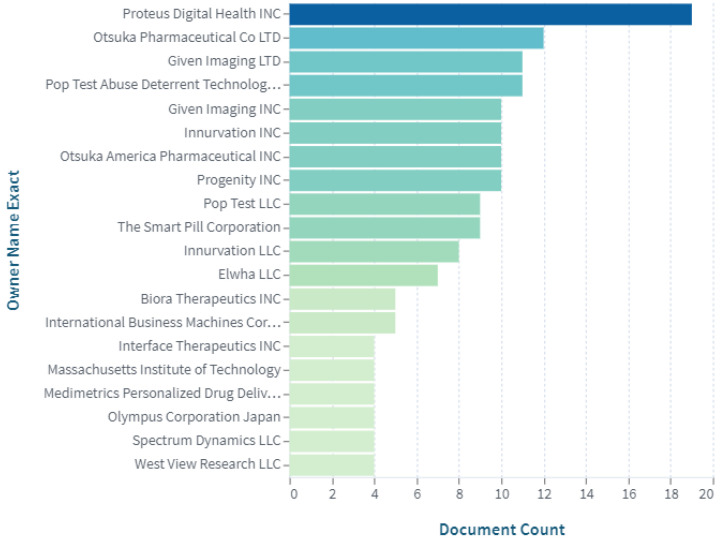
Top proprietors of patents in the field of digital pills with ingestible sensors (https://www.lens.org, accessed on 30 July 2022).

Figure 3 shows the principal owners of the patents in the field of digital pills with an ingestible sensor. Proteus Digital Health Inc. (Redwood City, CA, USA), Otsuka Pharmaceutical Co., Ltd. (Tokyo, Japan), Given Imaging Ltd. (Yokneam, Israel), Pop Test Abuse Deterrent Technology LLC (Cliffside Park, NJ, USA), Given Imaging Inc. (Duluth, GA, USA), Innurvation Inc. (Glen Burnie, MD, USA), Otsuka America Pharmaceutical Inc. (Rockville, MD, USA), Progenity Inc. (San Diego, CA, USA), Pop Test LLC (Cliffside Park, NJ, USA), The Smart Pill Corporation (Buffalo, NY, USA), and others are among the key companies committed to expanding the digital pill market size through innovative development.

**Figure 4:**
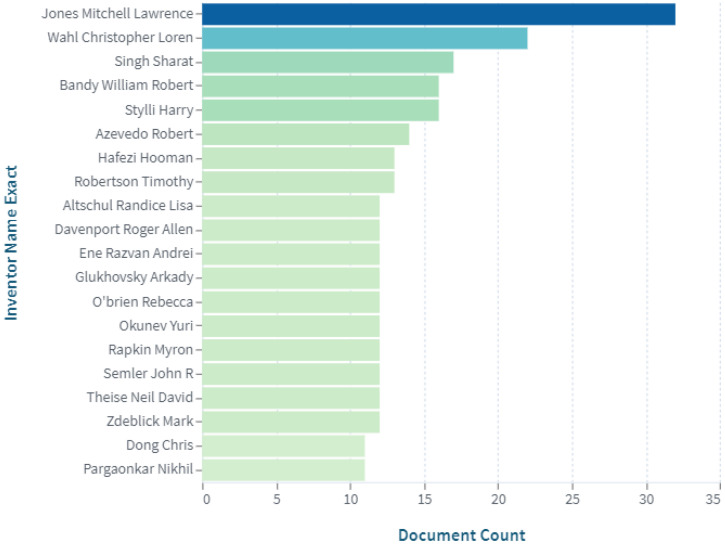
Top inventors of patents in the field of digital pills with ingestible sensor (https://www.lens.org, accessed on 30 July 2022).

The top 20 inventors involved in the creation of digital pills with ingestible sensors are shown in Figure 4. Their inventions are in the fields of mobile clinical monitoring, smart drug delivery, and endoscopy diagnostics. Mobile clinical monitoring and smart drug delivery make use of digital pills with sensors, and can be applied to treat a different range of diseases in a wide range of patients. A capsule endoscopy can help to diagnose problems with gastrointestinal peristalsis, such as persistent constipation, nausea, acid reflux, gastroparesis, colon cancer, and others. It should be noted that the digital pills with ingestible sensors are currently quite expensive, and the regulations for government approval of new products are strict. Within such a framework, scientists are actively working in the domain to bring effective and safe digital pills to the healthcare market.

The analysis (Figure 3) revealed that Proteus Digital Health (Redwood City, CA, USA) is one of the leading companies creating ingestible sensor systems for medication adherence. Proteus’ patented digital pill system allows for the personalization of treatment methods with wide potential benefits in various therapeutic areas. A digital ingestion tracking system—a sensor known as an “Ingestible Event Marker”—made by Proteus Digital Health was used to create the drug Abilify MyCite by Otsuka Pharmaceutical Company (Tokyo, Japan).

Abilify MyCite is an atypical antipsychotic equipped with an ingestible sensor for the treatment of adults with schizophrenia, the acute treatment of adults with manic and mixed episodes associated with bipolar I disorder, and for use as an adjunctive treatment for depression in adults. A serious problem associated with psychotropic drug treatment is adherence to the treatment plan. The side effects of antipsychotics are often debilitating, including weight gain, sexual dysfunction, nausea, and vomiting, and may cause people to change their own doses or stop taking the medication.

Aripiprazole, the primary chemical component of Abilify, was discovered by Otsuka Pharmaceutical in 1988, received FDA approval as an antipsychotic in 2002, and has been accessible as a generic drug since 2015.

The Abilify My-Cite (aripiprazole tablets with sensor), a drug–device combination product of aripiprazole tablets for oral administration integrated with an Ingestible Event Marker sensor, received the FDA’s first approval of a digital medicine system in 2017.

The four major components of Abilify MyCite interact using Bluetooth technology: the medication, which contains the active ingredient aripiprazole; a sensor that transmits a signal to a patch worn on the “lower edge of the rib cage”; a smartphone app; and an online portal. The 1-mm-sized sensor is built into the tablet. It is made of cuprous chloride (copper), magnesium, and silicon and releases a signal to the patch when it encounters stomach acid. When it comes in contact with the stomach acid, the magnesium and cuprous chloride within the sensor react to activate and power the device, and communicate a signal to the patch that is tracking ingestion. This information is then transmitted to the smartphone app [9].

The developed digital pills, such as Abilify MyCite, allow for the clinical monitoring of the treatment of patients with depression.

In order to improve the scientific and practical approaches to the management of scientific research in the process of digital pills with an ingestible sensor, an analysis was carried out of Proteus Digital Health and Otsuka Pharmaceutical’s patent strategy for the digital pill, Abilify MyCite (Table 1).

The performed analysis revealed that Abilify MyCite is protected by 32 US patents. This digital pill with an ingestible sensor has six hundred and seventy-one patent family members in forty-one countries.

As a result of the patent research, the patenting of a pharmacologically active ingredient aripiprazole and the technologies for its production, methods of treatment, as well as pharma-informatics systems and ingestible event marker systems, was established.

The collected data of the present review indicate the prospects and demand for digital pills with ingestible sensors in the global pharmaceutical market.

Abilify MyCite is under patent protection until 2030–2033. Licensing, which provides information about the process, is one of the ways to scale and accelerate the global long-term production of this digital drug. It is extremely important that the patent holders have the ability to control the effectiveness and quality of digital pills with ingestible sensors.

Proteus Digital Health is a company creating innovative digital health products and once had a huge valuation of USD1.5 billion. However, the company was unable to complete a USD100 million investment round in 2019. In the bankruptcy proceedings in 2020, a US affiliate of Otsuka purchased the technological assets of Proteus for USD15 million [42].

On the one hand, the rational management of intellectual human capital is extremely important in the development of digital pills. It is noted that the development of the digital pills with ingestible sensors, the Abilify MyCite technology, was expensive, and it was necessary to retain the best specialists. On the other hand, an assessment of medical technology is also very important. The average monthly cost of a generic version of Abilify is USD500 to USD800, according to GoodRx. The original digital pills with ingestible sensors, such as Abilify MyCite, cost more than USD1600.

In order to reduce the unpredictable rising costs of digital pills, it is imperative to perform comparative studies of the clinical effectiveness when discussing new treatment approaches, and to identify clear advantages over the medicines that are already used in clinical practice.

According to the ClinicalTrials.gov website, several clinical trials have been completed using the technology of digital pills with ingestible sensors. A number of digital pill clinical trials are ongoing.

The analysis of the patent landscape made it possible to identify the main therapeutic areas in which digital pills with ingestible sensors have proved themselves to be applicable. The following sections summarize the patented digital pills with ingestible sensors in the treatment of various pathological conditions.

**Diseases of the nervous system.**

Adherence is especially difficult in patients with serious mental illnesses, such as schizophrenia, schizophrenia-like disorders, and bipolar I disorder, with estimates of nonadherence as high as 50%. Adequate medication is essential to reduce the risk of major adverse outcomes in this population, such as psychosis, symptom recurrence, poor social functioning, hospitalizations, and suicide attempts [43].

As already mentioned above, a striking example of an active innovative strategy for creating digital pills with ingestible sensors is the tactics of Proteus Digital Health, Inc. (Redwood City, CA, USA). The inventions by this company are widely used in neurology and psychiatry. The company was formerly known as Proteus Biomedical, Inc. and changed its name to Proteus Digital Health, Inc. in July 2012.

The system, with a conductive element, an electronic component, and a partial power supply in the form of different materials, is described in patent US7978064 by Proteus Biomedical, Inc. The system is turned on when it comes into contact with a conducting liquid, since this completes the power supply and creates a voltage potential. To create a distinctive current signature, the electrical component regulates the conductance between the different materials. The system can be employed in a wide range of applications, such as ingestible event markers, ingestible identifiers, and pharmaceutical compositions with pharma-informatics capabilities.

The applicants of patent US8114021 Proteus Biomedical, Inc. disclosed a body-associated receiver, which may be external or implantable. Furthermore, the systems and procedures for using a receiver to coordinate with the dosage distribution systems are described.

The inventors of patent US8258962 (Proteus Biomedical, Inc., Redwood City, CA, USA) reported multi-mode communication ingestible event markers and systems, and the methods of using the same. The ingestible event marker consists of an integrated circuit component and upper and lower electrodes and is configured so that when it comes into contact with the stomach fluid, the current flows through the integrated circuit and causes one or more functional blocks in the circuit to emit a detectable signal. The upper and lower electrodes are made of different materials.

The pharma-informatics system is demonstrated in patent US8674825 (Proteus Digital Health, Inc., Redwood City, CA, USA). The automatic detection and identification of the pharmacological compounds actually delivered into the body is a significant new therapeutic tool provided to the clinicians by the present invention. This novel information system and device have numerous applications. Medication delivery, batch, and dose correlation to a physiological response can be achieved when employed in conjunction with other medical sensing equipment. In this way, the clinician may then create the best pharmacotherapeutic regimens. In the cases of accidental and other overdoses, the healthcare professional will be able to establish how far the intake has progressed and how many tablets are involved.

The development of a better pharma-informatics system (patent US 8945005, Proteus Digital Health, Inc., Redwood City, CA, USA) with a highly regulated identifier activation, where the signal produced by the identifier would be independent of the specific environment, such as stomach contents, the target site where activation is sought, represents another area of interest.

The effectiveness and safety of the above inventions of Proteus Digital Health were studied in clinical trials: NCT01804257 in patients (*n* = 28) with bipolar disorder or schizophrenia [44]; NCT03568500 in patients (*n* = 44) with schizophrenia, schizoaffective disorder, or first episode psychosis [45]; and NCT02091882 in patients with serious mental illness (*n* = 30) [46].

The clinical trials of Proteus Digital Health’s digital pills with ingestible sensors confirmed their high therapeutic efficacy, favorable safety profile, and ability to significantly improve patients’ quality of life. However, further post-marketing clinical trials are needed, including more patients with a high level of evidence.

**HIV/AIDS.**

HIV is one of the major global public health problems. Increasing access to effective HIV prevention and treatment allows patients to improve their health [47].

Proteus Digital Health inventions are widely used to monitor adherence in HIV-infected patients.

The digital health feedback system of Proteus (patents US7978064, US8114021, US8258962, US8545402, US8674825, US8718193, US8847766, etc.) was investigated in the NCT02797262 clinical trial for monitoring and increasing adherence to antiretroviral therapy in HIV-infected patients (*n* = 130), 18 years or older with sub-optimal adherence [48].

Moreover, the Proteus digital health feedback system is planned to be evaluated in the following clinical trials: in NCT02891720 to confirm ingestion of oral FTC/TDF as pre-exposure prophylaxis and to monitor adherence in HIV-negative YMSM (*n* = 100) [49]; in NCT04418037 in hospitalized individuals (*n* = 30) living with HIV to support ARV adherence [50]; and in NCT03693040 to collect information about patients (*n* = 100) taking their oral antiretroviral of TDF/FTC (Truvada) for HIV prevention [51].

The company etectRx (Gainesville, FL, USA) created an ingestible event marker (the ID-Cap^TM^ System), the technology of which is disclosed in the patent US9743880 B1 [52]. An electronic tag with an antenna and a receiver/transmitter placed on a pill capsule is part of a system and method for tracking a patient’s compliance with a medication regimen. A reader placed outside the body can detect the tag’s presence and location. The company will employ this technology in the NCT04065347 clinical trial to evaluate the relationship between adherence to antiretroviral therapy and HIV drug concentrations in people (*n* = 212) living with HIV (PLWH) who are taking tenofovir alafenamide [53].

The ID-Cap^TM^ System (made by etectRx, Gainesville, FL, USA) with Emtricitabine/Tenofovir (TDF/FTC) was investigated in an NCT03842436 clinical trial among MSM (*n* = 15) with substance use, to monitor PrEP adherence [54].

Since patient adherence to a medication therapy protocol is essential to preventor avoid costly consequences for the patient or the community, compliance monitoring using digital pills also offers important benefits in the prevention and treatment of HIV.

**Pain control.**

Another medical area with a wide use of digital pills with ingestible sensors is pain relief for various pathological conditions. Pain is a cause of passivity and depression. The somatic consequences of both acute and chronic pain are numerous, and sometimes catastrophic. Fever, while also being a protective, biologically appropriate reaction of the body, can at the same time cause a number of negative consequences [55].

The digital pills allow doctors to confirm that a patient actually took the painkillers within the predetermined time frame that they were scheduled to be delivered to them. This is a crucial tool for preventing the unintentional sale of medications that have not yet been consumed.

Tremeau Pharmaceuticals, Inc. (Concord, MA, USA) specializes in offering non-opioid pain relievers to well-defined patient populations with high unmet needs. The company has created digital pain management pills to treat pain more effectively and with fewer adverse effects.

The patent application US2021244672 A1 by Tremeau Pharmaceuticals Inc. (Concord, MA, USA) relates to digital pills in the discussed context [56]. An ingestible product, configured to be swallowed by a patient, comprises: a drug portion that requires a risk evaluation and mitigation strategy plan; and a wireless sensor portion configured to transmit sensor data concerning the drug portion to a remote device. The ingestible product is chosen from a group consisting of: a cyclooxygenase-2 Selective NSAIDs; and a D2 receptor antagonist.

Opioid analgesics are a critical treatment for those patients with advanced-stage pain from cancer. Cancer patients must have pain control as a mandatory and integral element of their treatment. Along with this, there are risks of long-term side effects, including possible abuse and addiction, which is a concern with the long-term use of opioid analgesics in patients who have had cancer.

The Proteus FDA-approved ingestible sensor (patent numbers US7978064, US8114021, US8545402, US8674825, US87181923, US8847766, etc.) was examined in the NCT04194528 clinical study on cancer patients (*n* = 2) with metastatic disease who were experiencing uncontrollable pain. Due to COVID-19′s restriction on site enrollment and the extremely high difficulty in acquiring sites to participate in the study, only two participants were acquired. When the provider of the DMP and the related software filed for bankruptcy and was acquired by a business that had no interest in completing the trial, the study was terminated. Due to a lack of patient data, the investigators did not examine any of the objectives besides the telemedicine feasibility [57].

Celero Systems (Lincoln, MA, USA) is a company that developed an ingestible system to detect and reverse opioid overdose. Celero Systems, inventors of CA3149412 A1, disclosed an opioid overdose rescue device that includes an ingestible capsule [58]. A non-refillable medication dispenser containing an opioid antidote, and at least one sensor that is set up to detect at least one physiological parameter suggestive of an opioid overdose are contained within the ingestible capsule. The ingestible capsule also has a controller that is operationally connected to the medication dispenser and a minimum of one sensor. If one physiological parameter is found to be outside a threshold value or range, this indicates an opioid overdose was detected. The ingested device can release a rescue drug via a drug dispenser and send out alerts to the patient and/or a caregiver upon recognizing the physiological signs of an opioid overdose.

As a result, there are numerous significant therapeutic uses for the capacity to record with digital pills the consumption of a medicine or other actual exposure of the body to a medicine, including pain treatment. The patients are reassured that they are correctly taking their prescribed medicines, thanks to this monitoring capacity. The possibility of over-prescription of drugs is avoided by this information.

**Cardiovascular diseases.**

Digital pills in cardiology also provide the doctor with a precise dose–response curve that shows the patient’s response to a medicine and the time at which the pill was administered. These data can be used in a variety of ways. Thus, the doctor can identify, for example, which people do not respond to the medicine in the tablet. Such patients might be excluded from a study to gauge the therapeutic effectiveness of a specific medicine. This ensures that the trial will only include the participants who have a favorable response to the medicine in question. This development will improve pharmaceuticals’ efficacy and encourage patients to use less ineffective treatments. It might also be used in clinical trials to track the individuals who took their prescription and those who did not.

Arterial hypertension remains one of the most common diseases in the developed world. Arterial hypertension triggers a chain of functional and structural changes in vital organs (first of all, in the heart, kidneys, and cerebral vessels), eventually leading to end-stage disease and the death of the patient [59].

The researchers managed persistent hypertension while receiving chronic anti-hypertensive therapy using the Proteus digital health feedback system (patents US7978064, US8114021, US8258962, US8545402, US8718193, US8847766, etc.) in the NCT02553512 clinical trial (*n* = 151) [60]. The system kept track of when each tablet was taken, how many steps were taken each day, how long patients slept and how often they were up, as well as the circadian rhythm for activity and relaxation.

The invention of WO2018200691 A2 relates to lisinopril compositions with an ingestible event marker (Proteus Digital Health, Inc., Redwood City, CA, USA) [61]. The current disclosure offers a special material composition that combines electronic circuitry with battery-forming materials and particular lisinopril formulations to verify the delivery of the particular lisinopril formulations. The specific lisinopril formulations are administered orally, and the current novel composition of the matter overcomes the unpredictable nature of mixing different metals and salts with them, to create an electronic delivery system that produces its own electrical power from a partial energy source composed of different materials when exposed to a patient’s bodily fluids.

Thus, digital pills make it possible for doctors to titrate to the most effective dosages of cardiac medicines by reducing the side effects, such as fatigue of the heart muscle and rebound effects, among others, and changing the dosage and time for each individual patient.

**Diabetes.**

Diabetes mellitus is a severe medical and societal issue worldwide. Over the past ten years, the number of diabetes mellitus patients has more than doubled, surpassing 463 million by the end of 2019. The International Diabetic Federation predicts that by 2030, 578 million people will have diabetes, and by 2045, 700 million people will be affected by this disease [62]. Digital technologies and sensors are widely used in the field of diabetes care. 

The global goal of treating patients with type 2 diabetes is to reduce the cardiovascular risks. In the NCT02827630 clinical trial, scientists evaluated the efficacy of Proteus Ingestible Sensor (patents US7978064, US8114021, US8258962, US8545402, US8674825, US8718193, US8847766, etc.) to lower blood pressure and glycated hemoglobin in patients (*n* = 118) with uncontrolled hypertension and type 2 diabetes [63].

While the discovery of novel pharmaceuticals to treat a range of disorders has increased in recent years, many of them have restricted use because they cannot be administered orally. Numerous factors contribute to this, including poor oral toleration, which can lead to issues such as gastric irritation and bleeding; the breakdown/degradation of medicine components in the stomach; and poor, sluggish, or irregular drug absorption. Conventional alternative techniques, such as intravenous and intramuscular injection, have a number of drawbacks, such as the discomfort and risk of infection from a needle stick, the requirement and risks of maintaining an IV line in a patient for a prolonged period of time, and the need to use sterile techniques. Despite the use of a variety of drug delivery techniques, including implanted drug delivery pumps, these techniques nevertheless share many of the same problems as IV delivery because they call for the semi-permanent implantation of a device. In order to treat diabetes and other conditions that influence blood glucose homeostasis, it is necessary to develop new techniques for giving drugs and other therapeutic agents, particularly for the improved delivery of insulin and other therapeutic agents.

The inventors of patent CA 2840617 C (Rani Therapeutics LLC, San Jose, CA, USA) disclosed a therapeutic preparation comprising insulin for delivery into the lumen of the intestinal tract, using a swallowable drug delivery device. In certain implementations, the user may externally trigger the actuating mechanism to administer a medicine via RF, magnetic, or other wireless signaling means, known as an alternative to or supplement to the internally initiated drug administration [64]. Moreover, the inventors noted that a delivery device may be used to distribute a number of medications for the treatment of multiple disorders or for the treatment of a specific condition. A combination of protease inhibitors may, for example, be used to treat HIV/AIDS. 

In recent years, new technologies for embedding under the skin or attaching to it for continuously measuring sugar levels have come to the market. They are systems that can be connected to other compatible medical devices and electronic interfaces, such as insulin pumps, automated insulin dosing systems, blood glucose meters, and other gadgets used in the management of diabetes.

The small intestine’s glucose concentration can be measured using an ingestible device, according to the invention EP3108810 A1 (Valtronic Tech (Holding) Sa, Les Charbonnières, Switzerland) [65]. The ingestible gadget includes a retainer to keep it in place in the small intestine for a short data transmitter/receiver. The method of the invention includes the steps of activating the device, fixing the device in the small intestine via a retainer, measuring the data related to glucose concentration inside the small intestine via a glucose sensor, and transmitting the data related to the glucose concentration to a receiver located outside the small intestine, particularly for determining or predicting the glucose level in the subject’s blood stream.

Thus, along with the patenting of digital pills with ingestible sensors for the treatment of diabetes, there is active patent protection of glucose sensors for indicating the blood and small intestine glucose levels of a patient with diabetes mellitus, and for smart drug delivery.

**Gastroenterology.**

Digital pills with ingestible sensors are widely used in gastroenterology. Better treatment regimens are still urgently needed to treat gastrointestinal disorders, such as inflammatory bowel disease. These regimens must be able to deliver therapeutics to specific areas of the gastrointestinal tract, while minimizing or avoiding the negative effects of oral or other systemic administration [66].

Several of Progenity, Inc.’s (San Diego, CA, USA) patents disclose novel therapeutic strategies for inflammatory gastrointestinal disorders.

The inventors of WO 2018/112255A1 (Progenity, Inc., San Diego, CA, USA) described a method of treating gastrointestinal disease with an immunosuppressant that is delivered through an ingestible device [67]. According to the method, the release of the medicine is triggered by data from a sensor or detector. The immunosuppressant is selected from Cyclosporine, Tacrolimus, and the generic equivalents thereof.

Application WO 2018/112240 A1 (Progenity, Inc., San Diego, CA, USA) described the use of a TNF inhibitor to treat a gastrointestinal disease [68]. An ingestible housing with a reservoir that stores a pharmaceutical composition containing a therapeutically effective amount of a TNF inhibitor is part of a TNF inhibitor delivery system. A detector is connected to the ingestible housing and is programmed to detect when the ingestible housing is close to a specific disease site.

Progenity, Inc. (San Diego, CA, USA) disclosed a targeted drug delivery system for the Il-6r inhibitor [69], an Il-12/Il-23 inhibitor [70], and an Il-1 inhibitor [71], employing one or more sensors connected to the ingestible housing.

When treating or reducing the symptoms of various medical illnesses, the therapeutic medications may occasionally need to be injected into specific regions of the small or large intestine. This is more effective than giving the medicine orally. The therapeutic drugs, for instance, may bypass the stomach’s gastrointestinal tract entirely and be delivered directly to the small intestine. This would make it possible to deliver a higher dose at a specific location inside the small intestine.

Along this line, the Drug Delivery System (DDS), developed by the biotechnology company Progenity, Inc. (San Diego, CA, USA), is of interest for the treatment of the gastrointestinal (GI) tract-related conditions. The applicants of EP3197336 B1 (Progenity, Inc., San Diego, CA, USA) disclosed an electromechanical pill device with localization capabilities [72]. By utilizing the reflection characteristics of organ tissue and sporadic particles, the ingestible device, which contains optical illumination sources and detectors that work at a variety of different wavelengths, may distinguish the areas of the gastrointestinal tract. Based on a detected device position, the ingestible device may sample fluid or release medication. The DDS will be used to target disease in the GI tract in order to improve efficacy by raising localized medicine concentration and lowering systemic adverse effects.

The inventors (Olympus Corporation, Tokyo, Japan) of patent US 8021356 B2 reported a capsule medication administration system [73]. This capsule medication administration system consists of an external device, a capsule-type medical device, a drug retention section, a drug release section, and a communication section. The external device has an external communication section that transmits and receives signals with the capsule-type medical device.

It should be noted that the ingestible sensors also allow for the collection of images and the tracking of luminal fluid and each gut segment’s contents, including electrolytes, enzymes, metabolites, hormones, and microbial populations. Due to the increasing usage of smart phones with Internet connectivity, the users and physicians can readily view and assess online the data produced by this technology.

Many ingestible capsules with sensors have been created and are set up to take pictures from inside passages and cavities within a body, such as those passageways and cavities within the GI tract. These gadgets often have an enclosed digital camera along with illumination light sources. Batteries or an external inductive power transfer may be used to power the capsule. Furthermore, the capsule might have a radio transmitter and/or memory for transferring data to an external receiver outside the body [66].

For instance, the inventors (Given Imaging Inc. (Duluth, GA, USA)and The Smart Pill Corporation (Buffalo, NY, USA)) of application US 2012/0209083 A1 explored the method of using, and determining the location of, an ingestible capsule [74]. An extracorporeal receiver can receive signals from an ingestible capsule that senses and transmits physiological parameters from patients. When in operation, the capsule and receiver carry out the process of locating the capsule in real time inside a mammal tract. This process involves supplying the capsule, which has one or more sensors, ingesting the capsule, having the capsule transmit a signal, having the capsule receive a transmitted signal, and then locating where the capsule is in the tract in real time, based on the received signal. The value of one or more sensed parameters may also be indicated by the received signal.

*Hepatitis C.*

Due to scientific breakthroughs in the treatment of HCV infection, it has been possible to make significant progress in the therapy of this pathology and to actually translate chronic hepatitis C into the category of diseases that are completely cured [75]. 

Thus, the NCT03164902 clinical trial will evaluate the ability of Proteus digital pills to promote adherence and thus achieve a cure for hepatitis C in the patients (*n* = 253) at a high risk of not adhering to their hepatitis C therapy [76].

The Proteus digital health feedback system (patents US7978064, US8114021, US8258962, US8545402, US8674825, US8718193, US8847766, etc.) is multi-fold. 

An effective antiviral therapy with digital pills, leading to the eradication of HCV infection, reduces the risk of progression of the hepatic and extrahepatic manifestations of HCV infection, especially if treatment is carried out before the formation of liver cirrhosis.

Without a doubt, digital pills with ingestible sensors have the potential to provide enormous amounts of information in the field of gastroenterology, along with imaging capsules. The dosing devices for releasing medicines into the gastrointestinal tract via one or more sensors are also promising to improve the effectiveness of treatment.

**Oncology.**

Health care costs pose a major challenge to national economic welfare. As a result of the aging of the population, the implementation of expensive innovative medicines, methods of radiation therapy and surgery, and diagnostic tests, the cost of treating oncopathologies increases, which is not always justified.

In some cases, the effectiveness of innovative, more expensive pills may not be supported by medical evidence, resulting in increased costs without improving outcomes. In the US, the term “financial toxicity” has come into use as a means of describing the financial stress that now often accompanies cancer treatment and reduces quality of life [77].

The researchers conducting the NCT04088955 clinical trial will collect and analyze data on the use of ingestible sensors with capecitabine or supportive medications (Proteus patents US7978064, US8114021, US8258962, US8545402, US8674825, US8718193, US8847766, etc.) and a digital feedback system on medication adherence and data-driven optimization of therapy for cancer patients (*n* = 500) [78].

Thus, further research is needed that aims to confirm that digital pills are not only clinically effective and relatively safe for the treatment of oncopathologies, but will also reveal their cost-effectiveness and possess additional benefits compared to other medicines.

**Tuberculosis.**

Multi-resistant tuberculosis is still a significant problem in the field of public health. Without the emergence of new pills, which in combination with other medicines could be used to create shorter, more effective, and less toxic treatment regimens, the global epidemic of multi-drug resistant tuberculosis will continue to grow [79].

The Proteus digital health feedback system (patents US7978064, US8114021, US8258962, US8545402, US8674825, US8718193, US8847766, etc.) was used in the NCT01960257 study to collect information about patients (*n* = 92) taking their tuberculosis medications [80]. The participants favored the digital health feedback system over the directly observed therapy for supporting confirmed daily adherence to tuberculosis drugs during the continuation phase of the tuberculosis treatment.

Therefore, taking digital pills can crucially contribute to avoiding the development of drug-resistant infectious disease strains, which can happen when proper dosing regimens are not adhered to. These resistant strains enhance the transmission, morbidity, and mortality, while costing significantly more to treat or eradicate, sometimes by orders of magnitude.

**Transplantology.**

Organ transplantation is associated with numerous concomitant conditions that affect the cardiovascular and other body systems. Careful follow-up of patients throughout transplantation is necessary. These patients need long-term rehabilitation with the participation of many specialists. Nonadherence to immunosuppressants leads to worse outcomes [81,82].

The investigations focused on the efficiency of the Ingestible Event Marker (Proteus patents US7978064, US8114021, US8258962, US8545402, US8674825, US8718193, US8847766, etc.) combined with Myfortic (360 mg) in adult kidney transplant patients (*n* = 30) are conducted within the NCT01320358 study [83]. 

The Ingestible Event Marker, according to the participating scientists, is a promising new device that offers incredibly accurate assessments of drug intake and timing in the clinical care of kidney transplant patients.

The above-described digital pill patents are presented for the purposes of illustration and not of limitation. There are a number of other inventions in the field of digital pills. In the last two decades, the scientists, in collaboration with industrial companies, have been activated in the search for effective and safe digital pills with ingestible sensors.

An analysis of the patent landscape showed that mobile clinical monitoring is widely used for treatment in the fields of mental health, HIV/AIDS, pain control, cardiovascular diseases, diabetes, oncology, tuberculosis, and transplantology. The devices for smart drug delivery and endoscopy diagnostics are patented in the field of gastroenterology to a greater degree. A detailed digital pill technology is presented in the descriptions of the patents. The description of the patent discloses the essence of the invention clearly and completely, so that it can be carried out by a person skilled in the art. However, the patent owner may also protect the technology as a trade secret.

It should be noted that digital pills are a new development both for pharmacy and medicine, which will be widely used in the future due to their advantages and in line with the global general digitalization trends. The key to their successful implementation is to ensure efficiency and safety, quality, and affordability, as well as compliance with ethical aspects in medical practice. Ensuring the continuous monitoring of the safety use of digital pills and identifying the potential side effects will help reduce and manage the risks associated with their use.

There is no doubt that the implementation of digital pills with ingestible sensors into the healthcare system is promising. Further large-scale comparative randomized clinical trials evaluating the efficacy and safety of digital and non-digital forms and meta-analysis data are needed. Nevertheless, it can be confidently predicted that soon all of these developments will come to fruition; progress cannot be stopped.

## 3. Materials and Methods

By the end of July 2022, search studies were conducted using Internet databases, such as the European Patent Office, the United States Patent Office, the United States Food and Drug Administration (FDA, Orange Book), the Lens database, Google Scholar, and ClinicalTrials.gov (accessed on 30 July 2022).

The following keywords were used in the patent search: ingestible sensor; digital pill; smart pill; and their combinations.

The International Patent Classification and Cooperative Patent Classification were utilized in conjunction with the title, abstract, and claims fields as search terms. When looking for information about patents, keywords are helpful. However, a lot of information is linked to many keywords and their synonyms, which makes analysis time-consuming and challenging. The use of language-neutral patent classification enables focusing the search, making it clearer and faster. Thus, the combined method using the keywords and codes of the International Patent Classification and the Cooperative Patent Classification improves the results of studies examining the patent landscape.

Digital products, such as subcutaneous and implantable sensors for continuous glucose monitoring systems, were considered to be outside the scope of this work and are not included in this study.

The analysis and systematization of the data made it possible to create the following search query: ((Q1 OR Q2 OR Q3) AND Q4). Q1, Q2, and Q3 are combinations of the keywords, and Q4 uses the International Patent Classification and Cooperative Patent Classification codes that match the digital pills.

The patent search results using the keywords: ingestible sensor; digital pill; smart pill; and their combinations, revealed 3101 patents and 1655 simple families.

Then, the 3101 patents were analyzed using the International Patent Classification and Cooperative Patent Classification codes (query Q4) to further filter and obtain relevant information. Their codes used in the search are shown in Table 2.

As result, the study altogether ultimately covered 291 patents, including 132 families. The yearly trends of the patent publications, the top 20 owners, the countries participating in innovations, and the key inventors were all studied. The most significant therapeutic application of digital tablets in medicine was identified.

Retrospective, logical, and graphic research methods and content analysis were used.

## 4. Conclusions

The patent landscape analysis shows an increase in the number of patents related to digital pills with ingestible sensors, which indicates the rapid progress and highly dynamic field of digital medicine technologies. The leaders in the number of patents issued are the United States, the European Patent Office, Canada, Australia, and China, which account for 72% of the total number of patents worldwide. The top 20 leading applicants were identified, whose inventions are related to the developments in the fields of mobile clinical monitoring, smart drug delivery, and endoscopy diagnostics.

The analysis revealed powerful patent protection for digital pills with an ingestible sensor, with often protection afforded by several patents in some countries, e.g., Abilify MyCite is protected by 32 US patents. This digital pill has six hundred and seventy-one patent family members in forty-one countries.

The following main areas of patenting digital pills with ingestible sensors were identified: treatment in the areas of mental health; HIV/AIDS; pain control; cardiovascular diseases; diabetes; gastroenterology (including hepatitis C); oncology; tuberculosis; and transplantology. The problems associated with the rapid implementation into medical practice are technical limitations, medical ethics, the legal framework, and thorough clinical trials of efficacy and safety.

Thus, the development of further scientific and practical approaches to the implementation of effective and safe digital pills will improve treatment outcomes, increase compliance, reduce hospital stays, provide mobile clinical monitoring, have a positive impact on treatment costs, and most likely become mainstream for most of the companies in the healthcare sector.

## Figures and Tables

**Table 1:** Analysis of Proteus Digital Health and Otsuka Pharmaceutical patent strategies for the digital pill Abilify MyCite.

US Patent No.	Patent Expiration	The Title of the Invention, the Owners
7053092	28/01/2022	5HT1a Receptor subtype agonist [10] Otsuka Pharmaceutical Co., Ltd. (Tokyo, Japan)
7978064	14/09/2026	Communication system with partial power source [11] Proteus Biomedical, Inc. (Redwood City, CA, USA)
8017615	16/06/2024	Low hygroscopic aripiprazole drug substance and processes for the preparation thereof [12] Otsuka Pharmaceutical Co., Ltd. (Tokyo, Japan)
8114021	21/06/2030	Body-associated receiver and method [13] Proteus Biomedical, Inc. (Redwood City, CA, USA)
8258962	25/11/2030	Multi-mode communication ingestible event markers and systems, and methods of using the same [14] Proteus Biomedical, Inc. (Redwood City, CA, USA)
8545402	27/04/2030	Highly reliable ingestible event markers and methods for using the same [15] Proteus Digital Health, Inc. (Redwood City, CA, USA)
8547248	18/12/2030	Implantable zero-wire communications system [16] Proteus Digital Health, Inc. (Redwood City, CA, USA)
8580796	25/09/2022	Low hygroscopic aripiprazole drug substance and processes for the preparation thereof [17] Otsuka Pharmaceutical Co., Ltd. (Tokyo, Japan)
8642760	25/09/2022	Low hygroscopic aripiprazole drug substance and processes for the preparation thereof [18] Otsuka Pharmaceutical Co., Ltd. (Tokyo, Japan)
8674825	09/04/2029	Pharma-informatics system [19] Proteus Digital Health, Inc. (Redwood City, CA, USA)
8718193	05/12/2029	Active signal processing personal health signal receivers [20] Proteus Digital Health, Inc. (Redwood City, CA, USA)
8759350	02/03/2027	Carbostyril derivatives and serotonin reuptake inhibitors for treatment of mood disorders [21] Otsuka Pharmaceutical Co., Ltd. (Tokyo, Japan)
8847766	29/03/2030	Pharma-informatics system [22] Proteus Digital Health, Inc. (Redwood City, CA, USA)
8945005	19/08/2029	Controlled activation ingestible identifier [23] Proteus Digital Health, Inc. (Redwood City, CA, USA)
8956288	06/07/2029	In-body power source having high surface area electrode [24] Proteus Digital Health, Inc. (Redwood City, CA, USA)
8961412	17/11/2030	In-body device with virtual dipole signal amplification [25] Proteus Digital Health, Inc. (Redwood City, CA, USA)
9060708	05/03/2029	Multi-mode communication ingestible event markers and systems, and methods of using the same [26] Proteus Digital Health, Inc. (Redwood City, CA, USA)
9089567	28/01/2022	Method of treating cognitive impairments and schizophrenias [27] Otsuka Pharmaceutical Co., Ltd. (Tokyo, Japan)
9119554	16/12/2028	Pharma-informatics system [28] Proteus Digital Health, Inc. (Redwood City, CA, USA)
9125939	28/07/2026	Carbostyril derivatives and mood stabilizers for treating mood disorders [29] Otsuka Pharmaceutical Co., Ltd. (Tokyo, Japan)
9149577	15/12/2029	Body-associated receiver and method [30] Proteus Digital Health, Inc. (Redwood City, CA, USA)
9258035	05/03/2029	Multi-mode communication ingestible event markers and systems, and methods of using the same [31] Proteus Digital Health, Inc. (Redwood City, CA, USA)
9268909	15/10/2033	Apparatus, system, and method to adaptively optimize power dissipation and broadcast power in a power source for a communication device [32] Proteus Digital Health, Inc. (Redwood City, CA, USA)
9320455	15/12//2031	Highly reliable ingestible event markers and methods for using the same [33] Proteus Digital Health, Inc. (Redwood City, CA, USA)
9359302	25/09/2022	Low hygroscopic aripiprazole drug substance and processes for the preparation thereof [34] Otsuka Pharmaceutical Co., Ltd. (Tokyo, Japan)
9387182	25/12/2023	Carbostyril derivatives and serotonin reuptake inhibitors for treatment of mood disorders [35] Otsuka Pharmaceutical Co., Ltd. (Tokyo, Japan)
9433371	15/09/2029	In-body device with virtual dipole signal amplification [36] Proteus Digital Health, Inc. (Redwood City, CA, USA)
9444503	19/11/2027	Active signal processing personal health signal receivers [37] Proteus Digital Health, Inc. (Redwood City, CA, USA)
9941931	04/11/2030	System for supply chain management [38] Proteus Digital Health, Inc. (Redwood City, CA, USA)
10441194	26/07/2029	Ingestible event marker systems [39] Proteus Digital Health, Inc. (Redwood City, CA, USA)
10517507	13/06/2032	Communication system with enhanced partial power source and method of manufacturing same [40] Proteus Digital Health, Inc. (Redwood City, CA, USA)
11229378	11/07/2031	Communication system with enhanced partial power source and method of manufacturing same [41] Otsuka Pharmaceutical Co., Ltd. (Tokyo, Japan)

**Table 2:** The International Patent Classification and Cooperative Patent Classification codes are used in the patent search.

Code	Meaning
International Patent Classification	
A61B5/00	Measuring for diagnostic purposes radiation diagnosis by ultrasonic, sonic, or infrasonic waves. Identification of persons
A61B5/07	Endoradiosondes
A61B5/145	Measuring characteristics of blood in vivo, e.g., gas concentration, pH-value measuring of blood pressure or blood flow non-radiation detecting or locating of foreign bodies in blood
A61K9/00	Medicinal preparations characterized by special physical form
Cooperative Patent Classification	
A61B5/073	Intestinal transmitters

## Data Availability

Data is contained within the article. Further queries addressed to the corresponding authors are welcome.
